# Antimicrobial Resistance Profiling of Pathogens from Cooked Donkey Meat Products in Beijing Area in One Health Context †

**DOI:** 10.3390/vetsci11120645

**Published:** 2024-12-12

**Authors:** Yiting Liu, Hongyun Duan, Luo Yang, Hong Chen, Rongzheng Wu, Yi Li, Yiping Zhu, Jing Li

**Affiliations:** 1 Equine Clinical Diagnostic Centre, College of Veterinary Medicine, China Agricultural University, Beijing 100193, China; sy20233051112@cau.edu.cn (Y.L.); lironyoung@cau.edu.cn (L.Y.); sy20233051128@cau.edu.cn (H.C.);; 2 Shenzhen Youlan Medical Technology Co., Ltd., Shenzhen 518102, China; 3 National Key Laboratory of Veterinary Public Health and Safety, College of Veterinary Medicine, China Agricultural University, Beijing 100193, China

**Keywords:** donkey meat product, *Proteus mirabilis*, *Klebsiella pneumoniae*, antimicrobial resistance, One Health

## Abstract

The prevalence of foodborne diseases has raised concerns due to the potential transmission of zoonotic bacterial pathogens through meat products. The objective of this study was to determine the occurrence and antimicrobial resistance (AMR) profiles of pathogenic bacteria in cooked donkey meat products from Beijing. Twenty-one cooked donkey meat samples were collected from different delis, subjected to homogenization, and analyzed for bacterial contamination. Molecular identification was performed through polymerase chain reaction (PCR) amplification and sequencing targeting the 16S rDNA gene. The antimicrobial susceptibility of the isolates was evaluated using the disk diffusion method. A total of forty bacterial isolates were identified, with *Proteus mirabilis* being the predominant species, followed by *Klebsiella pneumoniae* and *Novosphingobium*. Both *Proteus mirabilis* and *Klebsiella pneumoniae* exhibited high levels of resistance to several antibiotics, including penicillin, ampicillin, and erythromycin. This study’s findings underscore the public health risk posed by antimicrobial-resistant foodborne pathogens and emphasize the necessity for enhanced food safety surveillance within the One Health context.

## Simple Summary

Infections from foodborne pathogens can lead to gastroenteritis, which is commonly characterized by diarrhea, abdominal pain, and vomiting. Knowledge of the antimicrobial resistance patterns of foodborne pathogens is crucial for protecting public health. This facilitates the development of strategies to prevent the spread of resistant bacteria and ensures the effectiveness of treatments for foodborne diseases. This study examines the presence and antibiotic resistance of foodborne pathogens in donkey meat products from delis in the Beijing area. The results indicate a potential public health risk due to the presence of antibiotic-resistant bacteria in food products, emphasizing the need for enhanced food safety measures and antibiotic use oversight.

## 1. Introduction

Foodborne diseases, often attributed to a spectrum of pathogens including bacteria, viruses, fungi, and parasites, pose a significant threat to public health [1]. Bacteria are the predominant etiological agents, causing a myriad of diseases across human and animal populations [1]. Animal-derived meat and meat products, especially those consumed as ready-to-eat (RTE) items, are common vectors for zoonotic bacterial pathogens [2]. The processing of RTE food, which includes cooking, packaging, and storage, is critical in controlling pathogen prevalence throughout the food chain [1]. Nevertheless, the improper handling of these products can lead to contamination and introduce antimicrobial resistance (AMR) bacteria [3]. AMR genes can be transferred to humans through the consumption of meat and meat products containing resistant bacteria, emphasizing the utmost importance of the One Health approach to addressing AMR in public health [4]. Among various bacterial pathogens, *Staphylococcus aureus*, *Salmonella*, and *Listeria monocytogenes* are recognized as major causative agents of foodborne diseases, leading to substantial morbidity and mortality rates worldwide [5].

The increasing prevalence of foodborne disease outbreaks with a microbial etiology in China underscores the urgent need for stringent food safety monitoring [6]. The potential for these bacteria to transfer resistance genes to the human microbiome via the food chain represents a significant public health concern [7]. Donkey meat products are popular in China due to their quality proteins. However, there is a conspicuous lack of comprehensive data on the prevalence of pathogens and the antibiotic resistance phenotype in donkey meat products within China. This study aims to investigate the prevalence of bacterial pathogens and their susceptibility to antibiotics in cooked donkey meat samples from different delis in the Beijing area, which specialize in serving donkey meat products. The findings may contribute to the prevention and control of bacterial infection in donkey meat products and a more comprehensive understanding of food safety concerns from a One Health perspective.

## 2. Materials and Methods

### 2.1. Sample Collection

A total of 21 cooked donkey meat samples, comprising donkey burgers and stir-fried donkey meat, were collected via random sampling from 15 delis in different districts with notable sales volumes in the Beijing area. Each sample was aseptically excised using sterile instruments, encompassing both the surface and internal portions of the cooked donkey meat. After collection, the samples were transferred to the laboratory under preservation conditions at 4 °C.

### 2.2. Isolation and Identification

For the initial homogenization and pre-enrichment, 25 g of each sample was processed in 225 mL of modified tryptone soy broth (Qingdao Haibo, Qingdao, China) and incubated at 37 °C for 18 to 24 h. A 1 mL sample was diluted with 9 mL of sterile brain heart infusion broth (Qingdao Haibo, Qingdao, China) followed by thorough mixing. Subsequently, aliquots were spread onto MacConkey and Columbia blood agar plates (Qingdao Haibo, Qingdao, China) for further incubation at 37 °C for an additional 18 to 24 h to selectively isolate and identify bacterial colonies based on metabolic characteristics and to detect potential pathogens.

### 2.3. PCR Amplification and Identification

PCR amplification was conducted using universal 16S rDNA primers: the forward primer 27F (5′-AGAGTTTGATCCTGGCTCAG-3′) and the reverse primer 1492R (5′-TACGACTTAACCCCAATCGC-3′). The reaction mixture comprised 12.5 μL of 2 × Taq PCR Master Mix, 1 μL each of the forward and reverse primers, 0.5 μL of DNA template, and 10 μL of ddH_2_O. The thermal cycling protocol consisted of an initial denaturation at 95 °C for 5 min, followed by 30 cycles of 94 °C for 1 min, annealing at 55 to 58 °C for 1 min, and extension at 72 °C for 90 s, with a final extension at 72 °C for 10 min.

After the reaction, 10 μL of the PCR product was loaded on a 1.2% TAE-buffered agarose gel at 120 V for 25 min. The gel was visualized and documented using a UV imaging system with the DL 2000 DNA Marker (TIANGEN BIOTECH (BEIJING) Co., Ltd., Beijing, China) as a size reference. Positive PCR products were submitted for sequencing.

### 2.4. Antimicrobial Susceptibility Testing

The antibiotic susceptibility of bacterial isolates was assessed using the disk diffusion method, following the guidelines recommended by the Clinical and Laboratory Standards Institute (CLSI). The isolates were tested against a panel of 11 antibiotics: penicillin (PEM), ampicillin (AMP), ceftriaxone (CRO), cefazolin (CZ), tetracycline (TCN), amikacin (AMI), gentamicin (GEN), chloramphenicol (CHL), trimethoprim–sulfamethoxazole (SXT), ciprofloxacin (CIP), norfloxacin (NOR), erythromycin (ERY), and lincomycin (LCM). The selection of antibiotic categories and concentrations was determined in accordance with the “Performance Standards for Antimicrobial Susceptibility Testing; 4th Edition, M100” guidelines as stipulated by the CLSI. To ensure the reliability and reproducibility of the findings, each bacterial isolate was independently tested in triplicate.

## 3. Results

### 3.1. Bacteriologic Description 

A total of 40 bacterial isolates were obtained from the collected donkey meat samples. The genus *Proteus* showed the highest detection rate (40%), with *Proteus mirabilis* (*P. mirabilis*) (32.5%) being the predominant species (Table 1).

### 3.2. Antimicrobial Susceptibility Patterns

The isolates of *P. mirabilis* demonstrated a 100% resistance rate to penicillin, tetracycline, and lincomycin. Furthermore, the resistance to erythromycin and ampicillin was observed to be 84.62% and 61.55%, respectively (Table 2). Similarly, *Klebsiella pneumoniae* (*K. pneumoniae*) exhibited complete resistance to penicillin and erythromycin, with an 80% resistance rate to ampicillin (Table 2).

*Novosphingobium* also presented a 100% resistance profile against penicillin, ampicillin, and erythromycin (Table 3). These findings underscore the prevalence of AMR among the studied bacterial isolates.

## 4. Discussion

This study identified the presence of AMR bacteria in cooked donkey meat products from delis in the Beijing area. The bacterial isolates were predominantly *P. mirabilis*, followed by *K. pneumoniae* and *Novosphingobium*.

*P. mirabilis*, an opportunistic pathogen, is a well-known cause of nosocomial infections. Additionally, it is frequently implicated in urinary tract infections [8]. Its presence in donkey meat products, as identified in this study, raises concerns due to its potential to cause foodborne illness. *P. mirabilis* is known to inhabit various environmental niches, including soil and aquatic ecosystems, and is often found as a commensal organism in the gastrointestinal tracts of humans and animals [9]. *Proteus* species and *K. pneumoniae* have been isolated as thermotolerant bacteria from equids in India, which may survive in food-processing units and contaminate pasteurized products [10]. In a study by Liu Wei et al., from 2010 to 2019, there were 143 outbreaks of foodborne diseases in Haidian District, Beijing, with a total of 272 strains of pathogens detected, among which *Proteus* accounted for as high as 25.7%, becoming one of the main etiological agents of foodborne disease outbreaks in the area [11]. In a study conducted by Xiaobing et al., a total of 52 isolates of *P. mirabilis* were isolated from 178 cooked meat samples, with an isolation rate of 29.2% [12]. The presence of *P. mirabilis* in retail meat products could represent a pathway for the transmission of this pathogen to humans [13].

There was a marked increase in antibiotic-resistant *Proteus* spp. from 48.4% in 2011 to 74% in 2020 in hospital settings in Italy [14]. A particularly high resistance to macrolides, tetracyclines, fusidic acid, and mupirocin and a 77.6% resistance rate to aminopenicillins in *Proteus* spp. were detected in this research [14]. In the present study, a lower rate of 61.5% resistance to ampicillin was reported in the *P. mirabilis* isolates sourced from cooked donkey meat samples. This variance is probably due to the differing sources of the bacteria. Hospital-acquired strains are more frequently exposed to antibiotics, which likely contributes to increased resistance [14]. This highlights the importance of monitoring the prevalence and resistance patterns of this bacterium in food products from a One Health perspective. In research led by Delphine Girlich et al., *P. mirabilis* was found to be inherently resistant to several antibiotic classes including penicillin, macrolides, tetracyclines, and lincosamides [15]. This observation is consistent with the AMR patterns observed in the current study of *P. mirabilis*. This resistance is concerning as it restricts treatment options for infections caused by this bacterium. These findings underscore the alarming rise in antibiotic resistance among *P. mirabilis* strains, which not only poses a significant challenge to public health but also necessitates a comprehensive approach to antibiotic stewardship in both clinical and food production settings.

*K. pneumoniae*, recognized for its ability to inhabit the gastrointestinal tract of animals, has been identified as a common contaminant in various retail food products, including meats and vegetables [16]. This bacterium is a potential source of foodborne illness and can lead to extraintestinal infections in humans, posing a considerable public health concern [17]. In a study by Haryani et al., 32% of 78 street food samples from Malaysia were positive for *K. pneumoniae*, with isolates showing 100% resistance to ampicillin, erythromycin, and rifampicin and 80% to sulfamethoxazole [18]. Similarly, another study found *K. pneumoniae* in 7.5% (35/464) of cooked food samples, with the highest resistance noted for ampicillin at 92.3%, followed by tetracycline at 31.3% [19]. Additionally, the study indicated that cooking processes may not always be effective in eliminating bacteria [19]. The presence of *K. pneumoniae* in ready-to-eat cooked foods may suggest suboptimal hygienic practices during food handling or contamination after cooking. Possible sources of contamination include cross-contamination with raw products, meat juices, or other contaminated items or from handlers with inadequate personal hygiene. The thermotolerance of *K. pneumoniae* raises concerns, as it can survive in cooked foods, and its complete inactivation is not always guaranteed, even at temperatures up to 60 °C [10]. The resistance patterns also emphasize the importance of prudent antibiotic use and the implementation of stringent hygiene and handling protocols to prevent the spread of resistant strains.

The genus *Novosphingobium* encompasses a group of Gram-negative bacteria within the class Alphaproteobacteria, which was previously classified within the genus *Sphingomonas* [20]. Characterized by their metabolic versatility, these bacteria are known for their potential roles in the biodegradation of a spectrum of organic compounds [21]. In an investigation led by P. Papademas et al., high-throughput 16S rDNA sequencing revealed that *Sphingomonas* was the predominant genus in donkey milk from a farm in Cyprus, with a notable presence ranging from 17% to 49% [22]. Interestingly, while *Sphingomonas* was not detected in our analysis of donkey meat, we identified *Novosphingobium*, with analogous metabolic capabilities [22]. *Novosphingobium*’s metabolic diversity and role in the biodegradation of organic compounds are well documented, as is its isolation from multiple environments such as soil, water sediments, activated sludge, and even within plant tissues [23,24]. Importantly, this is the first report of *Novosphingobium* isolated from donkey meat, emphasizing the need for further investigation into its ecological distribution and potential impact on food safety. In one study, it was observed that colorectal cancer patients with reduced tumor tissue levels of *Caulobacter* and *Novosphingobium* showed enhanced long-term survival [25]. This may indicate the potential negative impact of *Novosphingobium* on human health. Regarding antibiotic resistance, Belmok et al. reported that *Novosphingobium terrae* exhibited resistance to penicillin, trimethoprim–sulfamethoxazole, and erythromycin [25], which is partially consistent with our findings. The ability of *Novosphingobium* to form biofilms and survive in chlorinated conditions raises concerns about its potential to harbor and protect other pathogens, including bacteria, which may contribute to the spread of antibiotic resistance [26]. It is worth noting that research on the antibiotic resistance of *Novosphingobium* is still limited, and further studies are required to fully understand the scope and implications of this issue. In addition, the impact of *Novosphingobium* on food safety is still uncertain.

The contamination of cooked meat products with pathogenic bacteria is a multifaceted issue, influenced by the introduction of bacteria during preparation, the sanitary conditions in the sampling site, and the prevalence of antibiotic resistance [5]. One of the primary factors identified is the presence of pathogenic bacteria such as *Proteus mirabilis*, *Klebsiella pneumoniae*, and *Escherichia coli*, which are known to thrive in environments lacking proper sanitation [5]. Furthermore, the risk of cooked meat contamination is increased by the conditions in the sampling site. Factors such as inadequate temperature control, the poor personal hygiene of food handlers, and unsanitary equipment can significantly contribute to the proliferation of these bacteria [27]. Our study indicates a high prevalence of *Proteus mirabilis* (32.5%) and *Novosphingobium* (15.0%), suggesting that the sampling site may have been compromised by poor hygiene practices. Additionally, the use of antibiotics in livestock can lead to AMR bacteria, which may contaminate meat during processing. Addressing these factors requires a multifaceted approach that includes stringent sanitation protocols, proper food handling education, and responsible antibiotic stewardship in livestock.

## 5. Conclusions

This study identified *Proteus mirabilis*, *Klebsiella pneumoniae*, and *Novosphingobium* as the dominant bacteria in cooked donkey meat products in the Beijing area, with notable resistance to common antibiotics including penicillin, ampicillin, and erythromycin. The findings underscore the need for further AMR monitoring and control in meat chains within the One Health context.

## Figures and Tables

**Table 1:** Bacteria and proportions of 40 isolates derived from donkey meat samples (n = 21).

Bacteria	Count	Ratio (%)
*Proteus mirabilis*	13	32.5
*Novosphingobium*	6	15.0
*Klebsiella pneumoniae*	5	12.5
*Escherichia coli*	3	7.5
*Proteus vulgaris*	2	5.0
*Streptococcus lutetiensis*	2	5.0
*Enterobacter hormaechei*	2	5.0
*Novosphingobium*	1	2.5
*Proteus vulgaris*	1	2.5
*Lysobacter*	1	2.5
*Enterococcus gallinarum*	1	2.5
*Macrococcus caseolyticus*	1	2.5
*Citrobacter freundii*	1	2.5
*Klebsiella oxytoca*	1	2.5

**Table 2:** Antimicrobial susceptibility results for *P. mirabilis* and *K. pneumoniae*.

Antibiotic Category	Antibiotics	*Proteus mirabilis*			*Klebsiella pneumoniae*		
		R (%)	S (%)	Sample Size	R (%)	S (%)	Sample Size
β-lactam antibiotics	PEM	100.0 (13)	0.0 (0)	13	100.0 (5)	0.0 (0)	5
	AMP	61.5 (8)	30.8 (4)	13	80.0 (4)	0.0 (0)	5
	CRO	8.3 (1)	66.7 (8)	12	-	-	-
	CZ	-	-	-	0.0 (0)	75.0 (3)	4
Tetracycline antibiotics	TCN	100.0 (12)	0.0 (0)	12	-	-	-
Aminoglycosides	AMI	-	-	-	0.0 (0)	100.0 (4)	4
	GEN	7.7 (1)	84.6 (11)	13	0.0 (0)	100.0 (5)	5
Chloramphenicols	CHL	46.2 (6)	38.5 (5)	13	0.0 (0)	100.0 (5)	5
Sulfonamides	SXT	38.6 (5)	46.2 (6)	13	0.0 (0)	80.0 (4)	5
Fluoroquinolones	CIP	7.7 (1)	84.6 (11)	13	0.0 (0)	100.0 (5)	5
	NOR	-	-	-	0.0 (0)	100.0 (4)	4
Macrolide antibiotics	ERY	84.6 (11)	15.4 (2)	13	100.0 (5)	0.0 (0)	5
Lincosamides	LCM	100.0 (12)	0.0 (0)	12	-	-	-

R (%) = (Number of strains resistant to the antibiotic/Sample size) × 100%. S (%) = (Number of strains sensitive to the antibiotic/Sample size) × 100%. AMK, amikacin; AMP, ampicillin; CHL, chloramphenicol; CIP, ciprofloxacin; CRO, ceftriaxone; CZ, cefazolin; ERY, erythromycin; GEN, gentamicin; LCM, lincomycin; NOR, norfloxacin; PEM, penicillin; SXT, trimethoprim–sulfamethoxazole; TCN, tetracycline.

**Table 3:** Antimicrobial susceptibility results for *Novosphingobium*.

Antibiotic Category	Antibiotics	*Novosphingobium*		
		R (%)	S (%)	Sample Size
β-lactam antibiotics	PEM	100.0 (6)	0.0 (0)	6
	AMP	100.0 (6)	0.0 (0)	6
	CZ	0.0 (0)	83.3 (5)	6
Aminoglycosides	AMK	0.0 (0)	100.0 (6)	6
	GEN	0.0 (0)	100.0 (6)	6
Chloramphenicols	CHL	0.0 (0)	100.0 (6)	6
Sulfonamides	SXT	50.0 (3)	50.0 (3)	6
Fluoroquinolones	CIP	0.0 (0)	100.0 (6)	6
	NOR	0.0 (0)	100.0 (6)	6
Macrolide antibiotics	ERY	100.0 (6)	0.0 (0)	6

## Data Availability

Data are contained within the article.
